# Bleeding Gastric Lipoma Resected by Endoscopic Submucosal Dissection

**DOI:** 10.7759/cureus.8909

**Published:** 2020-06-29

**Authors:** Adil S Mir, Varun Kesar, Mohamed Sageer, Douglas Grider, Vikas Chitnavis

**Affiliations:** 1 Internal Medicine, Carilion Clinic, Roanoke, USA; 2 Gastroenterology, Virginia Tech Carilion School of Medicine, Roanoke, USA; 3 Gastroenterology and Hepatology, Veterans Affairs Medical Center, Salem, USA; 4 Medicine/Gastroenterology, Virginia Tech, Roanoke, USA; 5 Basic Science Education, Virginia Tech Carilion School of Medicine, Roanoke, USA; 6 Pathology, Carilion Roanoke Memorial Hospital, Roanoke, USA; 7 Gastroenterology, Carilion Clinic, Roanoke, USA

**Keywords:** gastric lipoma, bleeding lipoma, endoscopic submucosal dissection

## Abstract

Gastric lipomas are slow-growing benign lesions of the stomach that are often detected incidentally. Most cases are asymptomatic but larger lesions may become symptomatic, thereby requiring treatment. Multiple endoscopic modalities have been used for resection in the past. We present the case of a 67-year-old patient who presented with upper GI bleeding secondary to a gastric lipoma, which was successfully resected by endoscopic submucosal dissection.

## Introduction

Gastric lipomas (GLs) are benign tumors composed of well-differentiated adipose tissue. GLs are rare and account for nearly 5% of the GI tract lipomas, about 3% of the benign tumors of the stomach, and less than 1% of all gastric tumors [1-3]. Within the stomach, lipomas are more commonly found in the antral region [2]. Most GLs are asymptomatic and found incidentally during endoscopic procedures or imaging for various other indications. However, GLs may become symptomatic by virtue of their size (usually more than 4 cm) and/or location [2]. Presenting symptoms may include abdominal discomfort or pain, dyspepsia, early satiety, nausea, vomiting, gastric outlet obstruction, or GI bleeding. Ulceration with bleeding is the most common manifestation of GLs and is seen in nearly 50% of the cases [2]. Whereas symptomatic subserosal lipomas have traditionally been managed by open procedures like enucleation, exploratory laparotomy, and gastrotomy, submucosal lipomas are usually amenable to endoscopic resection. We present a case of a large GL complicated by GI bleeding which was successfully removed by endoscopic submucosal dissection (ESD).

## Case presentation

**Figure 1:**
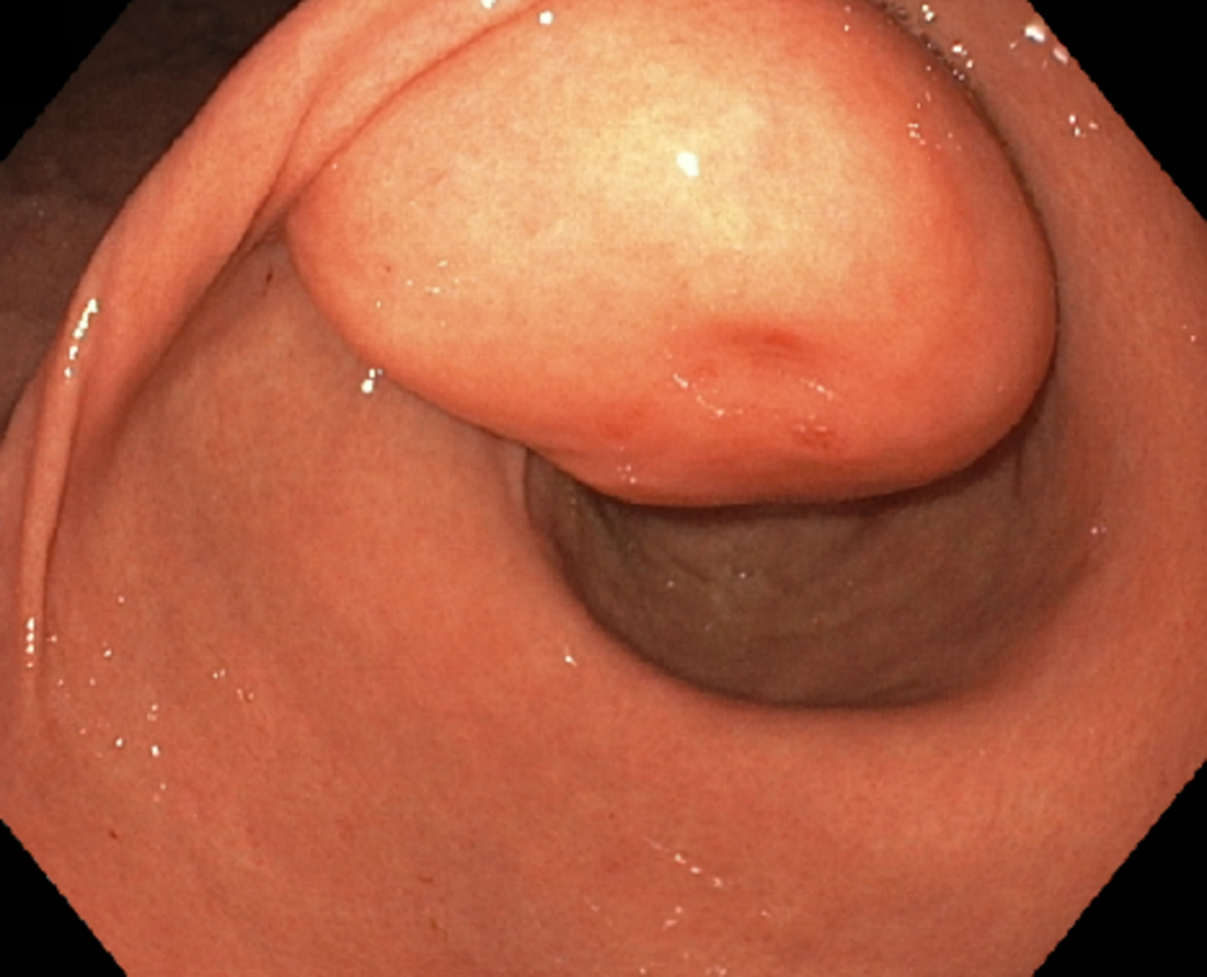
Gastric antral mass with a smooth surface below the incisura angularis

**Figure 2:**
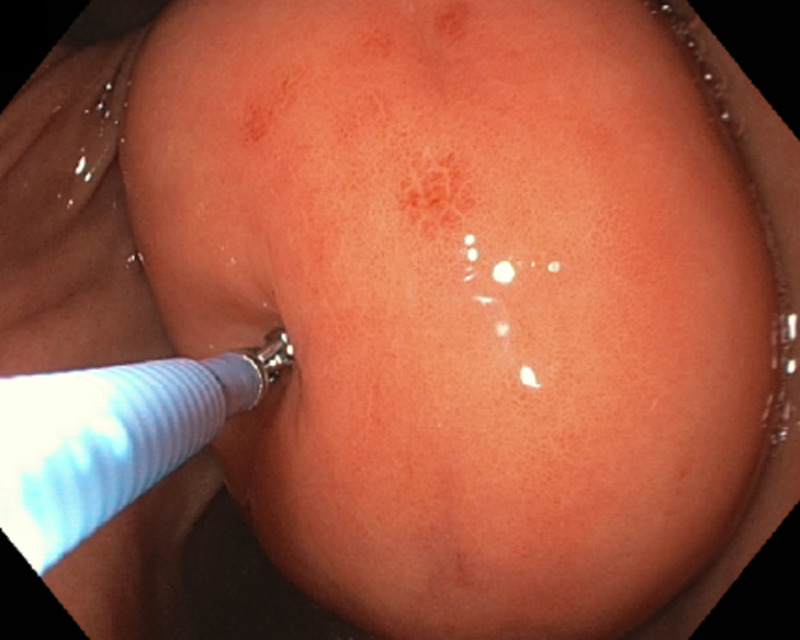
Pillow sign or cushion sign (application of mild pressure using a biopsy forceps causes indentation of the soft mass)

A 67-year-old male with a history of hereditary hemochromatosis presented for the evaluation of anemia. Workup included an esophagogastroduodenoscopy (EGD), which showed a large gastric antral submucosal mass (nearly 5 cm size) below the incisura angularis (Figure 1) with a positive ‘cushion’ or ‘pillow’ sign (Figure 2). No signs of bleeding were noted at this time.

**Figure 3:**
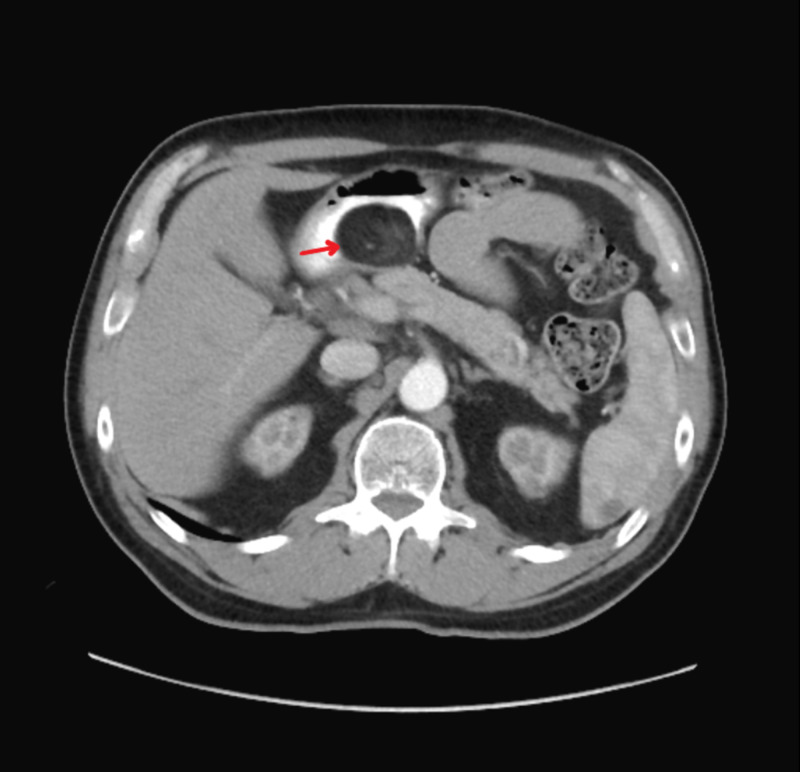
CT image showing a well-circumscribed mass in the lumen of the gastric antrum (arrow - axial view)

**Figure 4:**
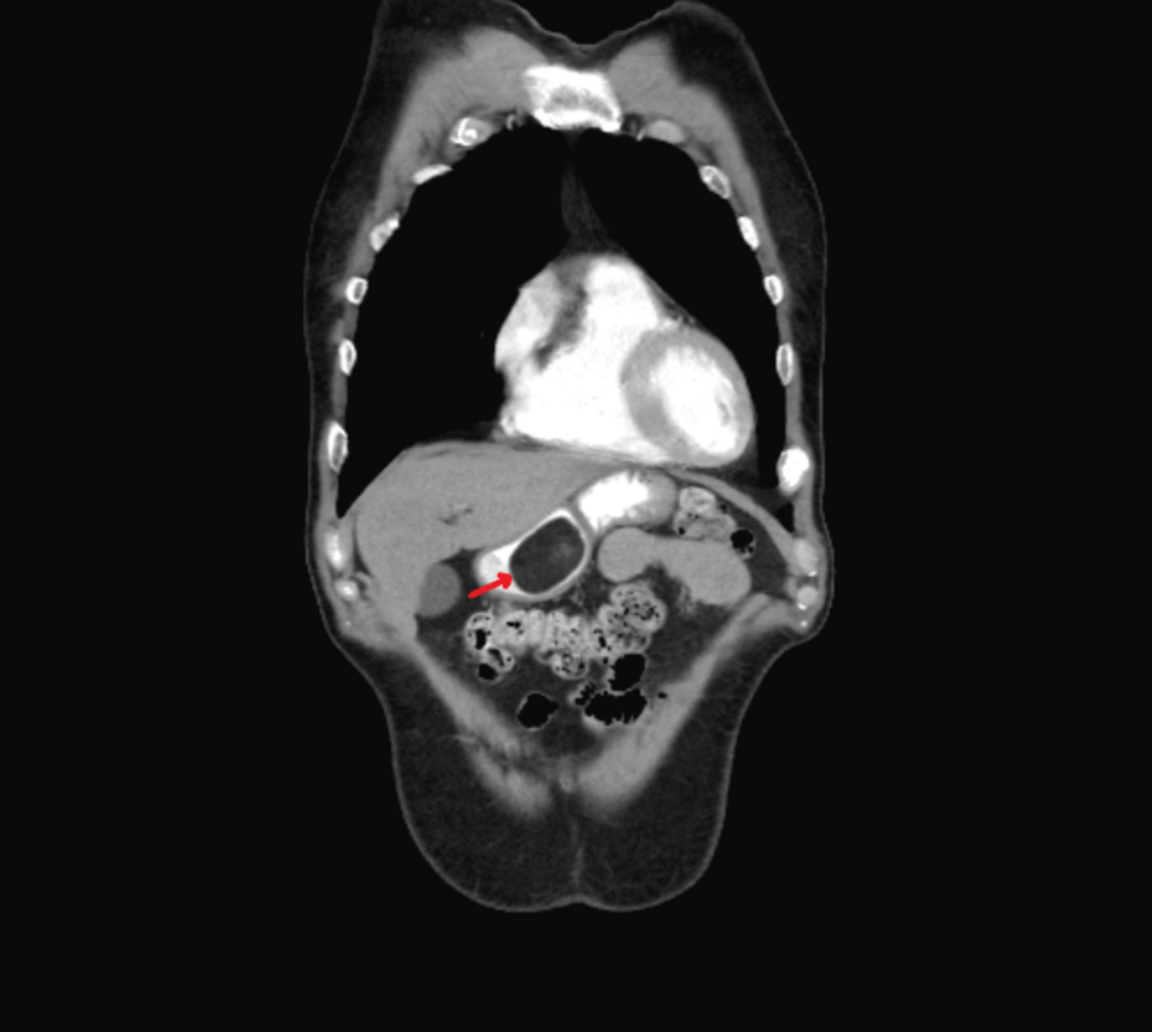
CT image showing homogeneous gastric mass with a well-defined border (arrow - coronal view)

CT scan showed a well-circumscribed mass within the lumen of the gastric antrum measuring 4.1 x 3.3 cm, likely representing a GL (Figures 3, 4), and the patient was discharged with outpatient follow-up. 

**Figure 5:**
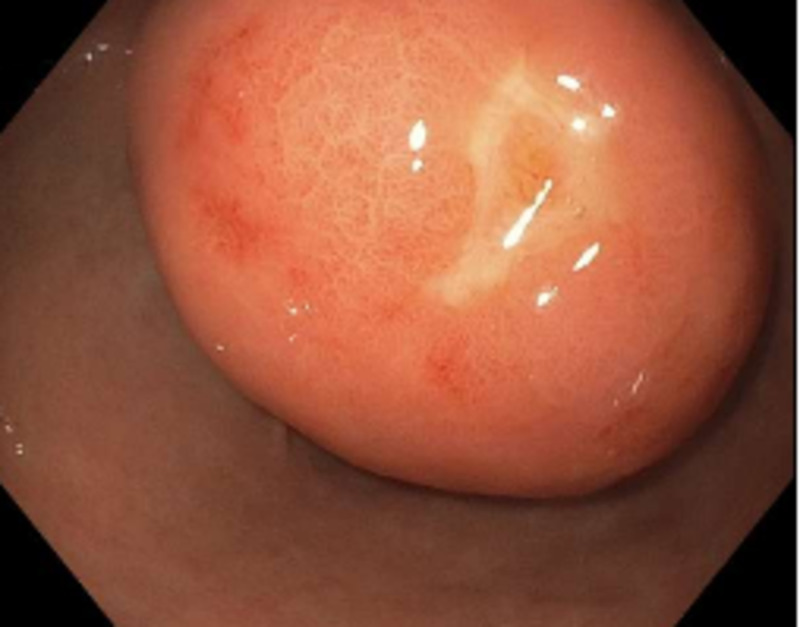
Gastric antral mass with a surface ulcer

Two years later, the patient presented with melena and coffee-ground emesis. Repeat EGD showed the previously detected antral mass with a surface ulceration (Figure 5). Endoscopic ultrasound (EUS) showed hyperechoic subepithelial lesion arising from the submucosa consistent with a lipoma. Subsequently, this lesion was unroofed exposing fat and bite biopsies were obtained that showed focal erosive gastritis (negative for Helicobacter pylori) and benign well-differentiated adipocytes in the submucosa. These findings are consistent with a submucosal lipoma pushing the mucosa into the stomach lumen with resultant irritant gastritis.

**Figure 6:**
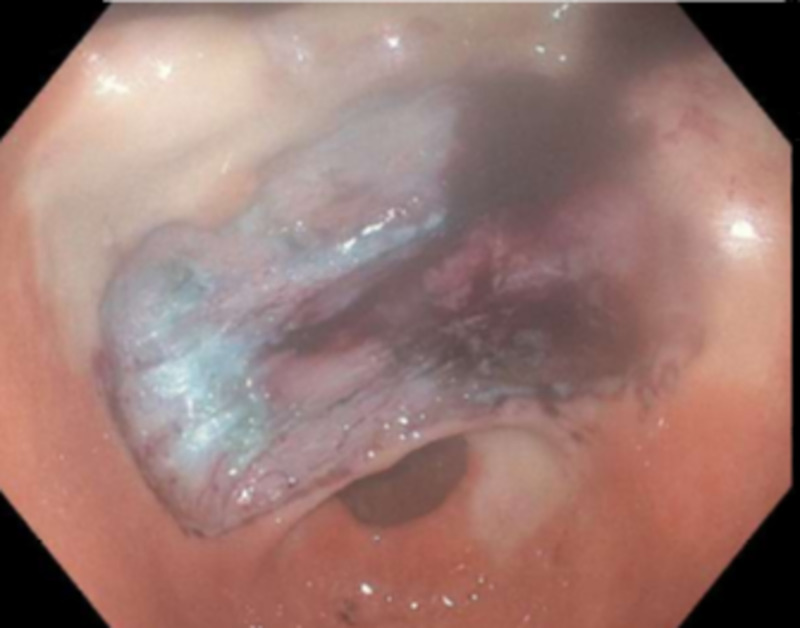
Endoscopic view of the gastric lipoma resection site after endoscopic submucosal dissection and cauterization

**Figure 7:**
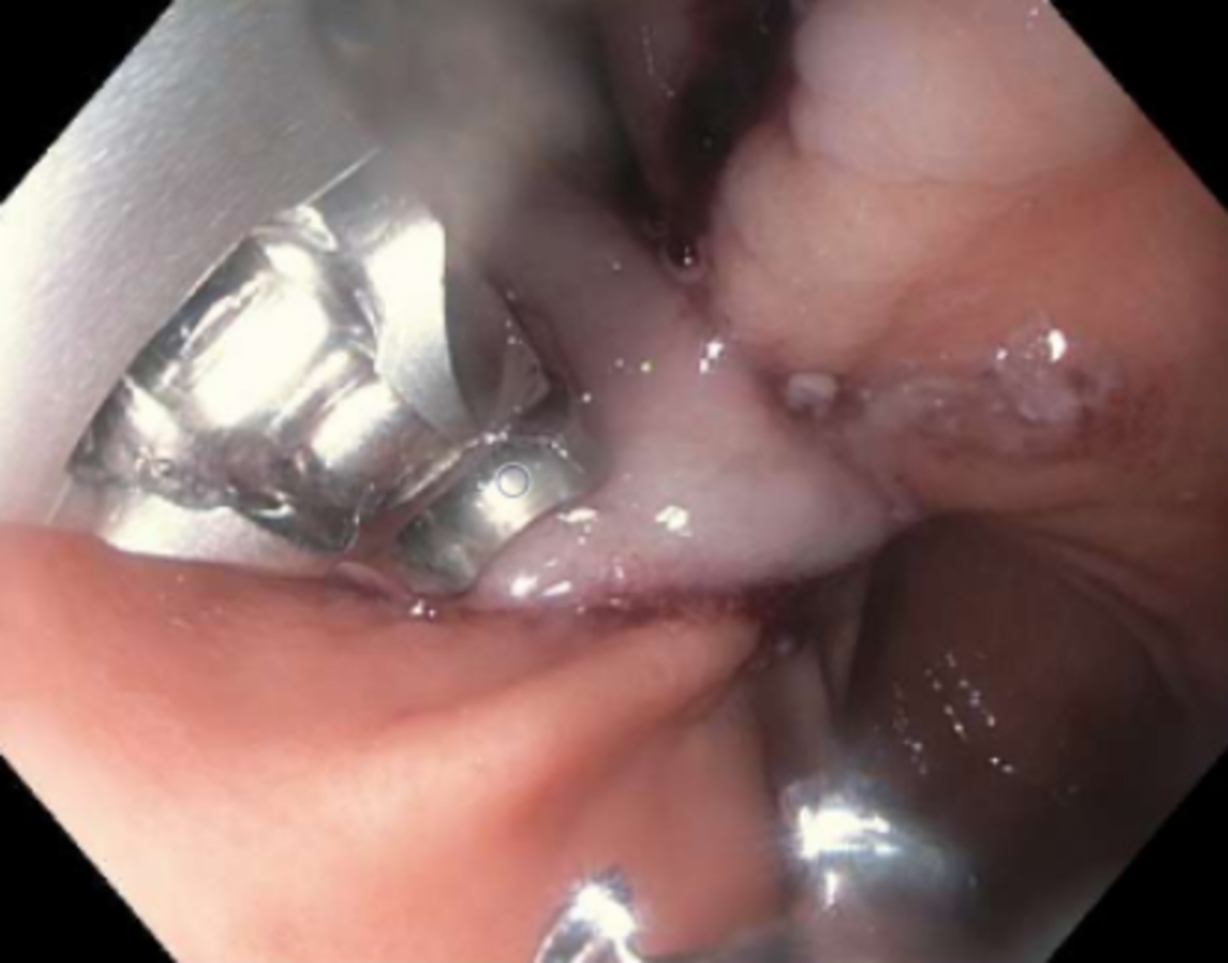
Endoscopic submucosal dissection site closed with single non-absorbable suture

**Figure 8:**
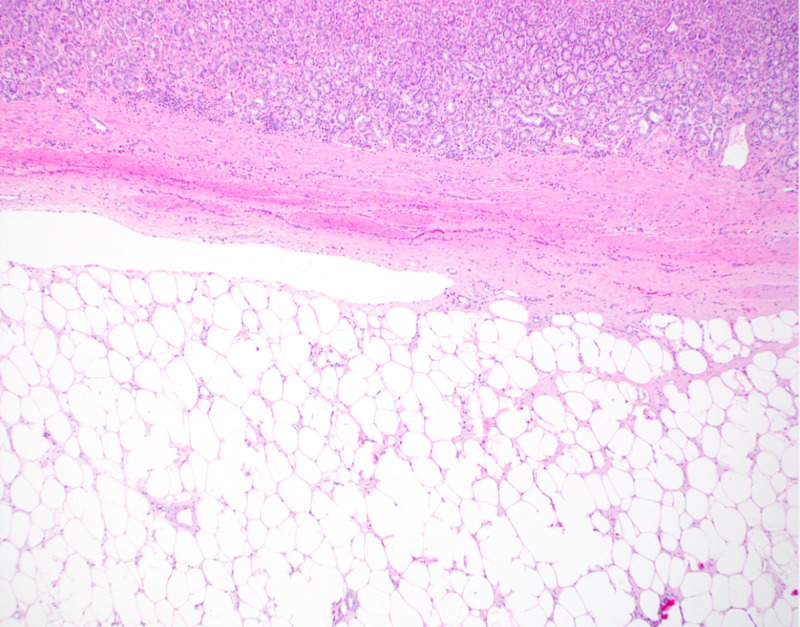
Benign adipocyte proliferation in the submucosa without extension into the mucosa but pushing the mucosa up (H&E, x40 magnification)

Subsequently, the patient underwent a repeat EGD with ESD and extirpation of a yellow-colored 7-cm submucosal lipoma followed by careful cauterization of the bleeders (Figure 6). The ESD site was finally closed with a single running non-absorbable suture (Apollo OverStitch, Apollo Endosurgery Inc, Austin, TX) (Figure 7). Histopathology confirmed a submucosal lipoma (Figure 8). There were no procedure-related complications and the patient was discharged with recommendations to follow up as outpatient. 

## Discussion

GLs are slow-growing benign tumors, more common in women as compared to men, and the average age at diagnosis in most cases is around 50 to 70 years [4]. Grossly, lipomas are yellowish appearing soft lesions with a smooth surface. The ‘cushion or pillow sign’ (Figure 2) (application of mild pressure using a biopsy forceps causes indentation of the soft mass), the ‘naked fat sign’ (extrusion of yellow fat from the mucosal breakdown spot on the lipoma after biopsy), and the ‘tenting sign’ (mucosa overlying the lipoma can be lifted and moved using a biopsy forceps) may support the diagnosis on gross examination [2,5]. CT imaging not only helps in diagnosis but also helps differentiate lipomas from other extramucosal tumors (like lymphoma or gastrointestinal stromal tumors). Typically, on CT imaging, lipomas appear to have well-defined borders with a homogeneous hypodensity of -70 and -120 Hounsfield units (HU) (Figures 3, 4) [2]. On EUS, lipomas appear as well-localized hyperechoic masses whereas on fat-suppressed MRI lipomas appear as uniformly localized hypodensities [3,4]. Histologic specimen to confirm the diagnosis can be obtained using fine needle aspiration cytology (FNAC) or sometimes biopsy, which may not be needed in most cases as the imaging and EUS features are characteristic [2].

As mentioned above, GI bleeding secondary to large GLs is not uncommon. In such cases, erosions and ulcerations are often noted during endoscopy. Ulcerated GLs are usually >4 cm in size, as in our case [6,7]. It has been debated that increased size of the lipoma may potentiate mucosal ischemia, leaving it vulnerable to the erosive effects of acid and mechanical effects of ingested food [4]. Although malignant transformation of GLs is rare, the possibility should always be borne in mind in case of large ulcerated masses that may present with obstructive features or bleeding. The diagnosis of liposarcoma can significantly alter the treatment decisions [2,6].

Asymptomatic GLs less than 2 cm in size that are incidentally diagnosed may only require observation, whereas symptomatic lesions that are >4 cm in size may need intervention [8]. GLs < 2 cm may be successfully treated with aspiration lumpectomy or strip biopsy [9]. Traditionally, large and symptomatic lesions were treated by surgery. However, with the advent of less invasive advanced endoscopic techniques, endoscopic resection is preferred option for submucosal GLs [10]. Endoscopic techniques used include snare resection, unroofing and enucleation, endoscopic mucosal resection (EMR), and ESD [10-12].

ESD of large GLs can be technically challenging, time consuming, and carries a significant risk of perforation. However, ESD has been used with success for the resection of subepithelial lesions including lipomas [10,12,13]. Also, due to a greater depth of resection, ESD is likely to be more effective in preventing recurrences as compared to other conventional techniques. Besides, ESD also allows for en bloc resection and secure hemostasis under direct endoscopic visualization [13].

## Conclusions

GLs are often incidentally diagnosed on endoscopy. EUS and imaging can help in diagnosis. Tissue diagnosis may be needed for confirmation or to rule out an underlying liposarcoma. The above case demonstrates that ESD can be used safely for the endoscopic resection of large submucosal GLs. The procedure requires more expertise and has a higher learning curve but is very effective in the definitive management of submucosal GLs.
